# Supplementary material for: Mathematical modelling indicates that lower activity of the haemostatic system in neonates is primarily due to lower prothrombin concentration
Source: Sci Rep. 2019 Mar 8;9:3936. doi: 10.1038/s41598-019-40435-7 (PMC6408458; doi:10.1038/s41598-019-40435-7)
Supplement: Supplementary file 1 — Supporting Material [file 41598_2019_40435_MOESM1_ESM.pdf]

SUPPORTING MATERIAL FOR "MATHEMATICAL MODELLING INDICATES THAT LOWER ACTIVITY OF THE HAEMOSTATIC SYSTEM IN NEONATES IS PRIMARILY DUE TO LOWER PROTHROMBIN CONCENTRATION"

IVO SIEKMANN[1,2], STEFAN BJELOSEVIC[3,4], KERRY LANDMAN[5], PAUL MONAGLE[3,6,7]
VERA IGNJATOVIC[3,7] & EDMUND J. CRAMPIN[1,2,5,8,9]

[1] Department of Applied Mathematics, Liverpool John Moores University, England
[2] Systems Biology Laboratory, Melbourne School of Engineering, University of Melbourne, Australia
[3] Haematology Research, Murdoch Childrens Research Institute, Melbourne, Australia
[4] The Sir Peter MacCallum Department of Oncology, University of Melbourne, Australia
[5] School of Mathematics and Statistics, University of Melbourne, Australia
[6] Department of Clinical Haematology, Royal Children Hospital, Melbourne, Australia
[7] Department of Paediatrics, University of Melbourne, Australia
[8] School of Medicine, University of Melbourne, Australia
[9] ARC Centre of Excellence in Convergent Bio-Nano Science and Technology, University of Melbourne, Australia

1. Variability between individuals within the same age group

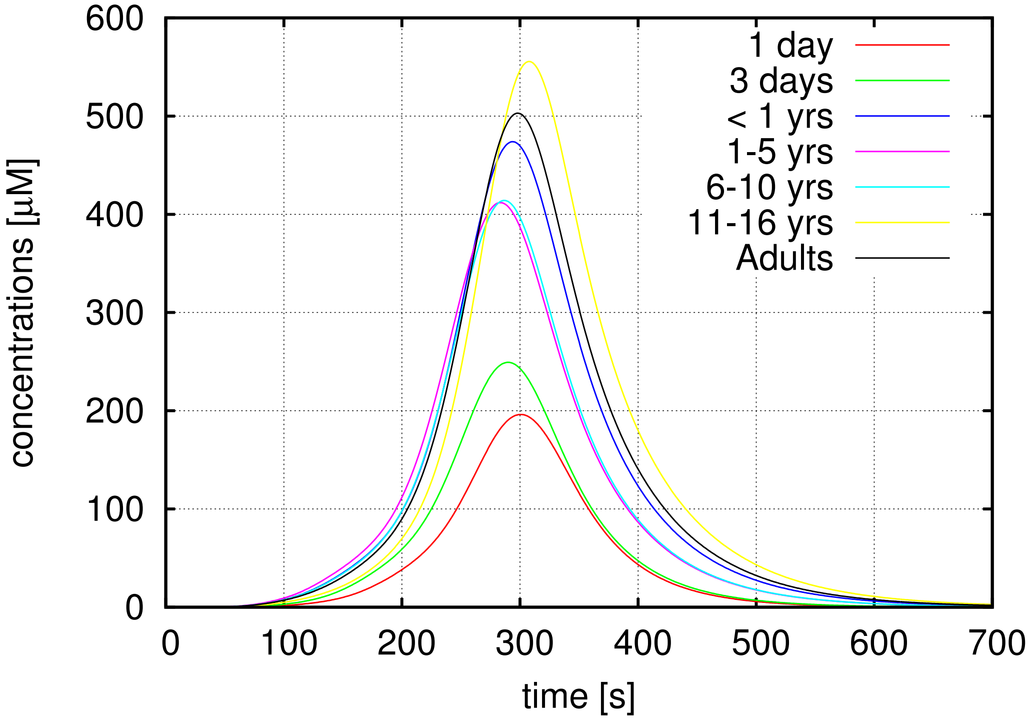

Figure S1. Age-dependent thrombin generation curves (TGC) calculated from the Hockin-Mann model [2] parameterised with the mean values of each age group from the data of Attard et al. [1]

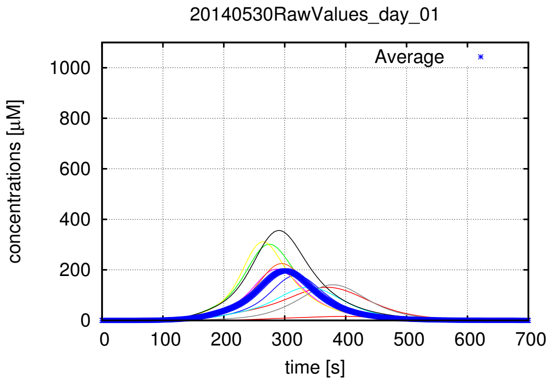

(A) day 1

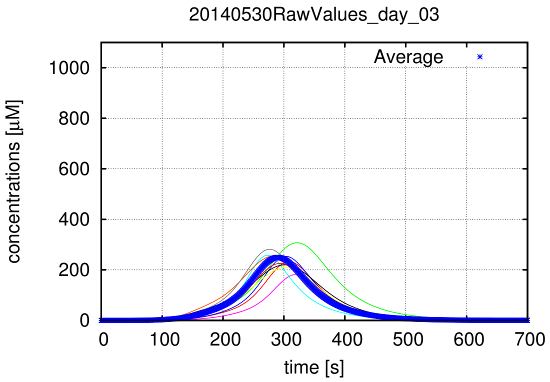

(B) day 3

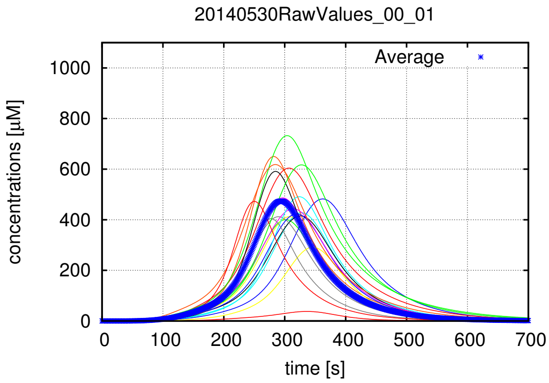

(C) less than 1 year

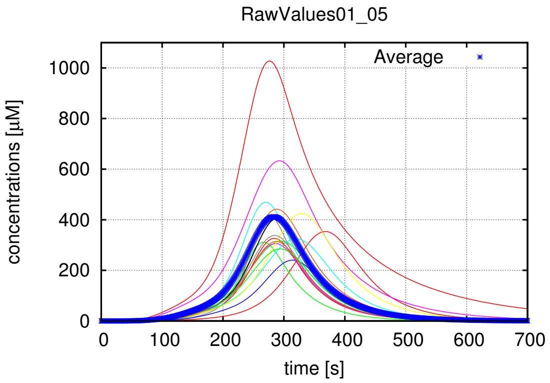

(D) 1-5 years

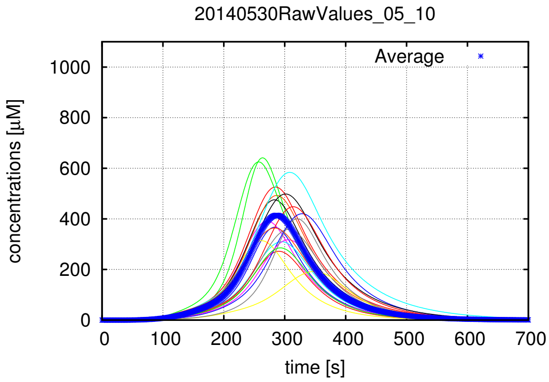

(E) 6-10 years

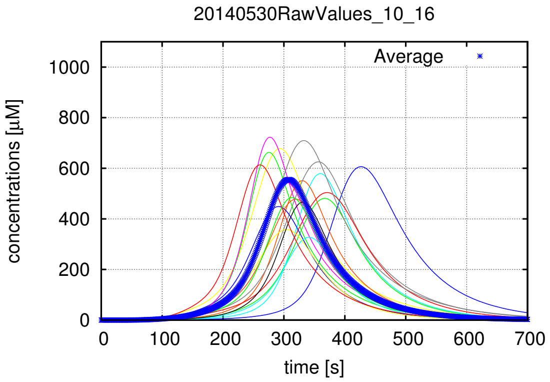

(F) 11-16 years

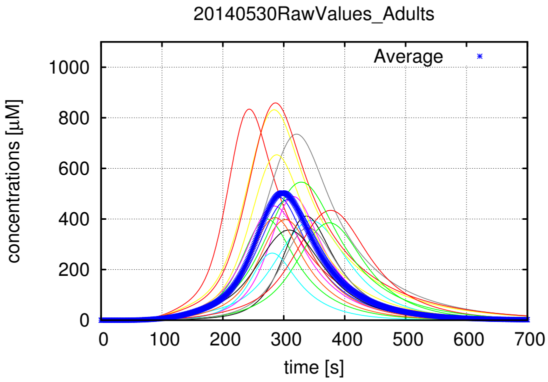

(G) Adults

FIGURE S2. TGCs calculated from the Hockin-Mann model [2] parametrised with data from individual patients [1].

2. SCATTER PLOTS FOR ALL COMBINATIONS OF FACTOR CONCENTRATIONS FROM [1]

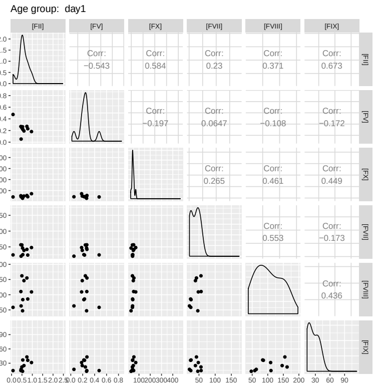

(A) day 1

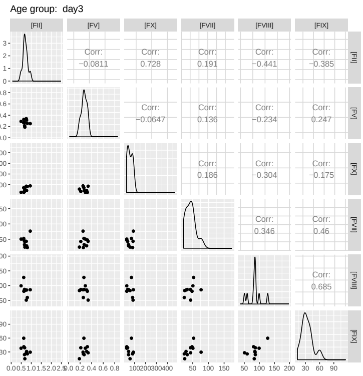

(B) day 3

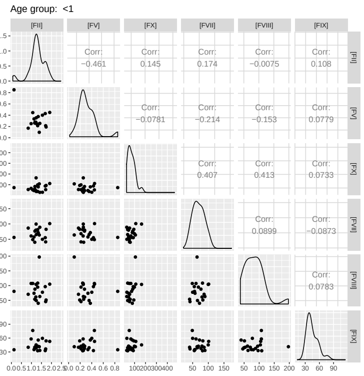

(C) less than 1 year

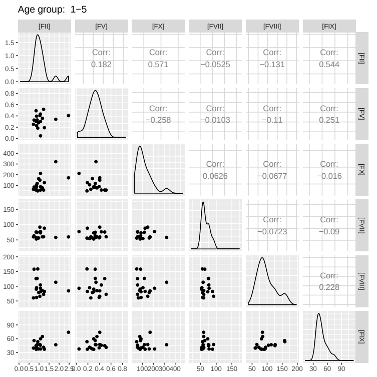

(D) 1-5 years

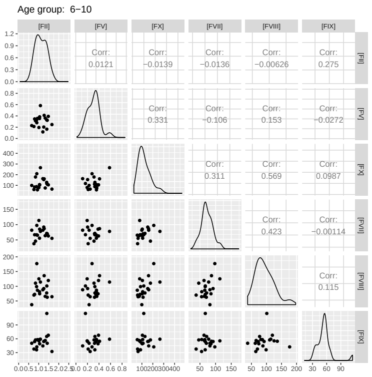

(E) 6-10 years

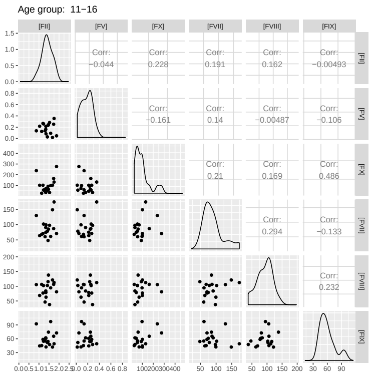

(F) 11-16 years

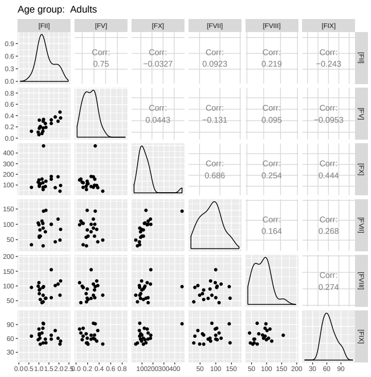

(G) Adults

FIGURE S3. Scatter plots for all combinations of coagulation factors from the data collected by Attard et al. [1].

3. Equations of the model by Hockin et al. [2]

$$\frac{d[TF]}{dt} = -k_2[TF][VII] + k_1[TF.VII] - k_4[TF][VIIa] + k_3[TF.VIIa]$$

$$\frac{d[VII]}{dt} = -k_2[TF][VII] + k_1[TF.VII] - k_5[TF.VIIa][VII] - k_6[Xa][VII] - k_7[IIa][VII]$$

$$\frac{d[TF.VII]}{dt} = +k_2[TF][VII] - k_1[TF.VII]$$

$$\frac{d[VIIa]}{dt} = -k_4[TF][VIIa] + k_3[TF.VIIa] + k_5[TF.VIIa][VII] + k_6[Xa][VII] + k_7[IIa][VII]$$

$$\begin{aligned}\frac{d[TF.VIIa]}{dt} &= +k_4[TF][VIIa] - k_3[TF.VIIa] - k_9[TF.VIIa][X] + k_8[TF.VIIa.X] - k_{12}[TF.VIIa][Xa] + k_{11}[TF.VIIa.Xa] - k_{14}[TF.VIIa][IX] + k_{13}[TF.VIIa.IX] \\ &\quad + k_{15}[TF.VIIa.IX] - k_{37}[TF.VIIa][Xa.TFPI] - k_{42}[TF.VIIa][ATIII]\end{aligned}$$

$$\begin{aligned}\frac{d[Xa]}{dt} &= -k_{12}[TF.VIIa][Xa] + k_{11}[TF.VIIa.Xa] + k_{22}[IXa.VIIIa.X] - k_{28}[Xa][Va] + k_{27}[Xa.Va] \\ &\quad - k_{34}[Xa][TFPI] + k_{33}[Xa.TFPI] - k_{38}[Xa][ATIII]\end{aligned}$$

$$\frac{d[IIa]}{dt} = +k_{16}[Xa][II] + k_{32}[Xa.Va][mIIa] - k_{41}[IIa][ATIII]$$

$$\frac{d[X]}{dt} = -k_9[TF.VIIa][X] + k_8[TF.VIIa.X] - k_{21}[IXa.VIIIa][X] + k_{20}[IXa.VIIIa.X] + k_{25}[IXa.VIIIa.X]$$

$$\frac{d[TF.VIIa.X]}{dt} = +k_9[TF.VIIa][X] - k_8[TF.VIIa.X] - k_{10}[TF.VIIa.X]$$

$$\frac{d[TF.VIIa.Xa]}{dt} = +k_{12}[TF.VIIa][Xa] - k_{11}[TF.VIIa.Xa] - k_{36}[TF.VIIa.Xa][TFPI] + k_{35}[TF.VIIa.Xa.TFPI] + k_{10}[TF.VIIa.X]$$

$$\frac{d[IX]}{dt} = -k_{14}[TF.VIIa][IX] + k_{13}[TF.VIIa.IX]$$

$$\frac{d[TF.VIIa.IX]}{dt} = +k_{14}[TF.VIIa][IX] - k_{13}[TF.VIIa.IX] - k_{15}[TF.VIIa.IX]$$

$$\frac{d[IXa]}{dt} = +k_{15}[TF.VIIa.IX] - k_{19}[VIIIa][IXa] + k_{18}[IXa.VIIIa] - k_{40}[IXa][ATIII] + k_{25}[IXa.VIIIa.X] + k_{25}[IXa.VIIIa]$$

$$\frac{d[II]}{dt} = -k_{16}[Xa][II] - k_{30}[Xa.Va][II] + k_{29}[Xa.Va.II]$$

$$\frac{d[VIII]}{dt} = -k_{17}[IIa][VIII]$$

$$\frac{d[VIIIa]}{dt} = +k_{17}[IIa][VIII] - k_{19}[VIIIa][IXa] + k_{18}[IXa.VIIIa] + k_{23}[VIIIa1.L][VIIIa2] - k_{24}[VIIIa]$$

$$\frac{d[IXa.VIIIa]}{dt} = +k_{19}[VIIIa][IXa] - k_{18}[IXa.VIIIa] - k_{21}[IXa.VIIIa][X] + k_{20}[IXa.VIIIa.X] + k_{22}[IXa.VIIIa.X] - k_{25}[IXa.VIIIa]$$

$$\frac{d[IXa.VIIIa.X]}{dt} = +k_{21}[IXa.VIIIa][X] - k_{20}[IXa.VIIIa.X] - k_{22}[IXa.VIIIa.X] - k_{25}[IXa.VIIIa.X]$$

$$\frac{d[VIIIa1.L]}{dt} = -k_{23}[VIIIa1.L][VIIIa2] + k_{24}[VIIIa] + k_{25}[IXa.VIIIa.X] + k_{25}[IXa.VIIIa]$$

$$\frac{d[VIIIa2]}{dt} = -k_{23}[VIIIa1.L][VIIIa2] + k_{24}[VIIIa] + k_{25}[IXa.VIIIa.X] + k_{25}[IXa.VIIIa]$$

$$\frac{d[V]}{dt} = -k_{26}[IIa][V]$$

$$\frac{d[Va]}{dt} = +k_{26}[IIa][V] - k_{28}[Xa][Va] + k_{27}[Xa.Va]$$

$$\frac{d[Xa.Va]}{dt} = +k_{28}[Xa][Va] - k_{27}[Xa.Va] - k_{30}[Xa.Va][II] + k_{29}[Xa.Va.II] + k_{31}[Xa.Va.II]$$

$$\frac{d[Xa.Va.II]}{dt} = +k_{30}[Xa.Va][II] - k_{29}[Xa.Va.II] - k_{31}[Xa.Va.II]$$

$$\frac{d[mIIa]}{dt} = +k_{31}[Xa.Va.II] - k_{32}[Xa.Va][mIIa] - k_{39}[mIIa][ATIII]$$

$$\frac{d[TFPI]}{dt} = -k_{34}[Xa][TFPI] + k_{33}[Xa.TFPI] - k_{36}[TF.VIIa.Xa][TFPI] + k_{35}[TF.VIIa.Xa.TFPI]$$

$$\frac{d[Xa.TFPI]}{dt} = +k_{34}[Xa][TFPI] - k_{33}[Xa.TFPI] - k_{37}[TF.VIIa][Xa.TFPI]$$

$$\frac{d[TF.VIIa.Xa.TFPI]}{dt} = +k_{36}[TF.VIIa.Xa][TFPI] - k_{35}[TF.VIIa.Xa.TFPI] + k_{37}[TF.VIIa][Xa.TFPI]$$

$$\frac{d[ATIII]}{dt} = -k_{38}[Xa][ATIII] - k_{39}[mIIa][ATIII] - k_{40}[IXa][ATIII] - k_{41}[IIa][ATIII] - k_{42}[TF.VIIa][ATIII]$$

$$\frac{d[Xa.ATIII]}{dt} = +k_{38}[Xa][ATIII]$$

$$\frac{d[mIIa.ATIII]}{dt} = +k_{39}[mIIa][ATIII]$$

$$\frac{d[IXa.ATIII]}{dt} = +k_{40}[IXa][ATIII]$$

$$\frac{d[IIa.ATIII]}{dt} = +k_{41}[IIa][ATIII]$$

$$\frac{d[TF.VIIa.ATIII]}{dt} = +k_{42}[TF.VIIa][ATIII]$$

References

[1] Attard, C., T. van der Straaten, V. Karlaftis, P. Monagle, and V. Ignjatovic (2013). Developmental hemostasis: age-specific differences in the levels of hemostatic proteins. *Journal of Thrombosis and Haemostasis 11*, 1850–1854.

[2] Hockin, M. F., K. C. Jones, S. J. Everse, and K. G. Mann (2002). A model for the stoichiometric regulation of blood coagulation. *Journal of Biological Chemistry 277*(21), 18322–18333.
